# The impact of early life experiences on inhibitory control and working memory

**DOI:** 10.3389/fpsyg.2024.1484424

**Published:** 2024-11-28

**Authors:** Eva Dydenkova, Francis McGlone, Larisa Mayorova, Elena Nikolaeva

**Affiliations:** ^1^Moscow Affective Touch Laboratory, Pushkin State Russian Language Institute, Moscow, Russia; ^2^Faculty of Science & Engineering, School of Life Sciences, Manchester Metropolitan University, Manchester, United Kingdom; ^3^Laboratory of Physiology of Sensory Systems, Institute of Higher Nervous Activity & Neurophysiology of Russian Academy of Science, Moscow, Russia; ^4^Laboratory of Experimental Neurology & Neuroimaging, Federal Research & Clinical Center of Intensive Care Medicine and Rehabilitology, Moscow, Russia; ^5^Developmental psychology & Family pedagogic department, Herzen University, Saint Petersburg, Russia

**Keywords:** working memory, inhibitory control, retrieval-based learning, early institutionalisation, children, adverse childhood experiences

## Abstract

**Introduction:**

Adverse childhood experiences (ACEs) are a general term for a large group of nonequivalent situations that have the potential to traumatise a child. This risk factor is caused by a sensitive period of brain development, which is based on myelination, creation of synaptic connections and pruning. Dramatic environmental events during this period, such as history of institutionalisation, can disrupt optimal developmental pathways, leaving biological scars for life.

**Methods:**

The focus of this study was to investigate the impact of institutionalisation on the development of inhibitory control and working memory in three groups of children matched for age (*n* = 130; 7.1 ± 2.0 years): (1) early institutionalised (*n* = 35; age of placement: 6.9 ± 10.6 months; duration of placement: 14.6 ± 10.4 months); (2) late institutionalised (*n* = 29; age of placement: 49.3 ± 30.6 months; the duration of placement: 16.0 ± 19.4 months); (3) never institutionalised (*n* = 66).

**Results:**

Results showed that the early institutionalised group had the lowest scores on tests of inhibitory control (*p* = 0.03), working memory (*p* = 0.03) and retrieval-based learning (*p* = 0.04), while the results of the group of late institutionalised children do not differ significantly from never institutionalised.

**Discussion:**

The existence of a sensitive period during the first 18 months of a child’s life is discussed, which determines the formation of the retrieval-based learning mechanism and of inhibiting ineffective experience, for which executive functions are responsible.

## Introduction

Adverse childhood experiences (ACEs) are a general term for a large group of nonequivalent situations (Berman et al., 2022) that have the potential to traumatise a child (Sheridan et al., 2022; Wade et al., 2020; Malave et al., 2022; Lund et al., 2020). This risk factor is caused by a sensitive period of brain development, which is based on myelination, creation of synaptic connections and pruning (Haynes et al., 2025; Stiles and Jernigan, 2010; Uylings, 2006). The consequence of these processes will be the formation of the child’s connectome responsible for cognitive functions. In addition, these processes are due to the interaction of the child’s genetic apparatus, controlled by environmental factors (Gao et al., 2019). Dramatic environmental events during this period can disrupt optimal developmental pathways, leaving biological scars for life (Malave et al., 2022; Hakamata et al., 2022; Lund et al., 2022). One of the variants of traumatic experience is the abandonment of the child by a parent, leading to a history of institutionalisation. There is currently a consensus regarding the negative impact of institutionalisation on child development (Anthony et al., 2019; Bakermans-Kranenburg et al., 2003; Beckett et al., 2006), formed on the basis of an understanding of the consequences outlined in attachment theory (Bowlby, 1951; Bowlby, 1953), the concept of sensitive periods in early ontogenesis (Walasek et al., 2024) and an analysis of the consequences of stressful events on children (Gunnar and Quevedo, 2007; McLaughlin et al., 2019).

According to attachment theory, early relationships, primarily with the mother, not only determine the child’s current state of development, but also have significant consequences for his future cognitive outcomes, self-regulation, and ability to establish close relationships both during childhood and in adulthood (Beckett et al., 2006; Bowlby, 1953; Sroufe, 2005; Bernier et al., 2010). The concept of sensitive periods (Voss, 2013; Robson, 2002; Nelson and Gabard-Durnam, 2020; Knudsen, 2004) describes the influences that regulate the formation of a certain function in a certain period of time and determine the final result of the development of this function (Gunnar and Quevedo, 2007; Bailes et al., 2024). Both the data obtained from attachment research (Santaguida and Bergamasco, 2024) and the results of the study of children at the psychophysiological level indicate that the first 18 months of life are a highly sensitive period for the formation of a child’s interactions with the social world (Nelson, 2001).

Moreover, this is confirmed by studies where attempts to correct cognitive and psychosocial impairments in the child’s development, as well as problems with establishing secure attachment, were made at different periods of time, and it was shown that the maximum benefits of such interventions were found if they were implemented before the age of 18 months (Bakermans-Kranenburg et al., 2008; Wright and Edginton, 2016; Nelson et al., 2023).

Among the many consequences of institutionalisation, the deficit in the child’s cognitive functions is one of the most difficult to restore (Merz et al., 2013; Peñarrubia et al., 2020; Wade et al., 2019). First of all, a decrease in IQ is noted, which is sometimes explained by a change in brain volume (Mackes et al., 2020; Bick and Nelson, 2017). While foster care significantly restores both psycho emotional development (Forbes and Gallo, 2017; McCall et al., 2016; McCall et al., 2016; Garvin et al., 2012; Jaffari-Bimmel et al., 2006) and intelligence (Sánchez, 2015; Sonuga-Barke et al., 2017; Nelson et al., 2007; Van Ijzendoorn et al., 2005), for some aspects of cognitive development the negative consequences of institutional experience persist long after placement in a foster family (Bakermans-Kranenburg et al., 2008; Wade et al., 2019). Here we focus on executive functions which refers to a family of top-down mental processes needed, as Diamond describes, “to take time to think before acting; meeting unanticipated challenges; resisting temptations; and staying focused” (Diamond, 2013; Friedman and Miyake, 2017). In other words, using executive functions is effortful because they are involved in changing a patterned, habitual behaviour to a new one, that is appropriate to the new circumstances. Three core executive functions are usually distinguished: inhibitory control, working memory and cognitive flexibility (Friedman and Miyake, 2017; McKenna et al., 2017; Miyake et al., 2000). Given that the formation of executive functions is associated with the development of the prefrontal cortex, and is therefore long-term and non-linear (Friedman and Robbins, 2022; Anderson, 2002; Lieberman, 2020), this also indicates a large window open to both positive and negative environmental influences.

But what are these influencing factors? There is currently no clear understanding of what else, besides the fact of placing a child in an orphanage, influences the process of restoring executive functions after placing a child in a foster family. First of all, this difficulty is explained by the complexity of organising studies involving institutionalised children. Children have very different life histories prior to placement in an orphanage: both pre-institutionalisation experiences (Hawk et al., 2018), reasons for institutionalisation (Berman et al., 2022; The St. Petersburg–USA Orphanage Research Team, 2005), and post-institutional experiences (Vreeland et al., 2020) vary greatly. This suggests that each child can have a unique life-path influenced by many different factors, however their influence is reduced by the creation of a certain group of institutionalised children. And although all researchers came to the same conclusion that the longer the institutionalisation and the later the foster care intervention, the more pronounced the deficits in inhibitory control and working memory (Colvert et al., 2008; McDermott et al., 2013; Loman et al., 2013; Merz et al., 2013; Pollak et al., 2010; Desmarais et al., 2012), there is still confusion with age and the duration of placement in an institution (Julian, 2013). The challenge of studying the effects of institutional history also relates to individual child differences in vulnerability and resilience to early adversity, as well as in the quantity and quality of these experiences.

A meta-analysis of over 30,000 children with early adversity found that psychosocial deprivation, but not threat, was associated with significant declines in both inhibitory control and working memory (Johnson et al., 2021). Children adopted between 4 and 60 months of age with prior institutional experience continued to make more errors on an inhibition task nearly a decade later than their biological peers (Lewis et al., 2024). Another study compared children who were institutionalised immediately after birth with children who grew up in foster care. Both groups performed worse on inhibition and memory tasks 8 years later than a never institutionalised group, and the effect of foster care was found only after controlling for prenatal factors (Bos et al., 2009). Stinson et al. (2024) assessed the state of inhibitory control in children at 8 years old, after they were adopted from an orphanage at age 3, finding that differences in executive functions persisted.

There is evidence that the long-term consequences of institutionalisation are more pronounced the earlier the child enters the orphanage. Moreover, there is an opinion that the risk of increasing social and psychological problems is not determined by gradual changes, but is abrupt, sharply increasing in a certain time range (Julian, 2013). The age at which significant changes occur is determined by the severity of the conditions of institutionalisation. And such a jump may occur at 6 months for Romanian children studied within the Bucharest project (Rutter, 2006) and 18 months for Russian children (Naumova et al., 2019). The presence of such a boundary effect was also noted in the study, which noted that staying in a blood family during the first 9 months after birth reduced the severity of the consequences of further stay in an orphanage (Hawk et al., 2018).

The hypothesis to be tested in this study is that there is a critical period for the formation of optimum working memory and inhibitory control within the first 18 months of a child’s life. This suggests that children who were placed in an institution during this period and then despite being placed in a substitute family will not achieve the parameters of working memory and inhibitory control that will be found in children who end up in an institution after this period. We make this assumption based on the relationship between these executive functions, the development of the prefrontal cortex, and the importance of the first years of life in the functional development of the prefrontal cortex (Jeong et al., 2024; Fox et al., 2010).

## Materials and methods

### Description of the sample

According to the Ministry of Education of the Russian Federation, the number of orphans in the country is decreasing (by 9% in 2 years), but the number of these children is still huge—358 thousand orphans in 2023 (this is 1.2% of all children). Most of them—322.6 thousand (90%)—live in substitute families, while the rest of the children (>30 thousand)—in children’s institutions. Although the official figures are decreasing, in reality there may be significantly more children in orphanages (~60 thousand). These statistics do not include children placed on the basis of a “parental application for temporary placement in an institution” either immediately after birth or at any stage of childhood. Officially, such children are placed in an institution for 6 months, but often the application is rewritten every 6 months. Formally, they retain the status of “family,” and they cannot be transferred to a substitute family, so the entire childhood of such “temporarily” placed children can be spent in the orphanage system.

Since there is no institution of foster family in Russia, we are going to use the term “substitute family” or “substitute care.” The term “substitute care” refers to a family of non-specialists in child rearing who accept a child for a fee and raise him or her until the age of 18. The study did not include adopted children, i.e., children who were taken into a family permanently. The official status of care for a child who has experienced institutionalisation (in Russia this is mainly adoption and substitute family) was not an inclusion criterion, but adopted families are often a difficult sample to access due to the secrecy of adoption. In addition, despite the fact that adoption, according to the Family Code, is a priority form of placement (including due to the lower risk of abuse), according to the Ministry of Education of the Russian Federation, the share of adopted children in Russia is falling every year and in 2023 it amounted to only 7% of family placements.

The study recruited 130 children, of whom 64 (years 7.5 ± 2.1) had histories of institutionalisation (months 15.2 ± 15.0) and at the time of the study lived in substitute families (months 48.6 ± 25.1). The comparison group consisted of 66 children (years 6.6 ± 1.8) living in families with biological parents and never having had the experience of institutionalisation. Children in the ever-institutionalised group were placed in an institution on average at the age of 16.4 ± 14.3 months (range 0–39 months). The samples of never institutionalised children and ever-institutionalised children did not differ by sex or the level of mothers’ education, but biological mothers were on average 10 years younger than the substitute mothers (Table 1).

**Table 1:** Characteristics of study sample.

Indicators		Never institutionalised (*n* = 66)	Ever-institutionalised (*n* = 64; 15.2 ± 15.0 months of institutionalisation)	Statistics
Sex	Boys	39	37	Pearson chi-square = 0.02; *p* = 0.88
	Girls	29	27	
Age of placement (months, M ± SD)		–	16.4 ± 14.3	–
Duration of placement (months, M ± SD)		–	15.2 ± 15.0	–
Staying in substitute care (months, M ± SD)		–	48.6 ± 25.1	–
Mother’s age (years, M ± SD)		36.1 ± 3.9	46.3 ± 6.4	*W* = 0.97; *p* = 0.05; *t* = −11.1; *p* < 0.001
Mother’s education level	Bachelor’s degree	11	15	Pearson chi-square = 0.93; *p* = 0.34
	Master’s degree	55	49	

### Intelligence level assessment

The level of intelligence within the normal range was the criterion for inclusion of subjects in the study sample. The level of nonverbal intelligence was assessed using Colored Progressive Matrices (RCPM) (Raven et al., 2008) for children up to and including 9 years of age and using Standard Progressive Matrices (RSPM) (Raven et al., 2008) for subjects up to and including 11 years of age. The actual level of intelligence was compared with the normative values for each age. Children with intelligence levels within the normal range participated in the study.

The study was approved by the Ethical Committee IRB 00011060 Herzen State Pedagogical University of Russia#1 protocol dated 11/27/2023.

## Methods

### Assessment of inhibitory control and working memory

To assess the development of inhibitory control and working memory, two computer tests were used, the software for which was installed on a laptop with the Windows 11 operating system. These programmes can be accessed for downloading and further use upon request sent to the corresponding author.

### Inhibition task

**Figure 1:**
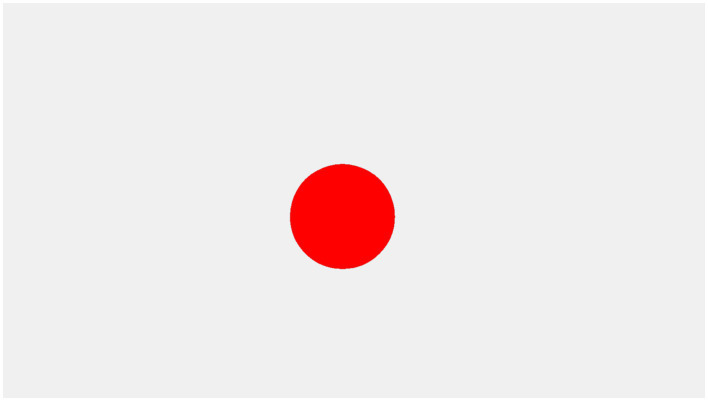
Screen shot of one of the series of the inhibition task.

Inhibitory control was determined using go/go and go/no-go paradigms (Vergunov et al., 2018; Vergunov and Nikolaeva, 2009; Nikolaeva et al., 2020). The task includes three series: one training and two experimental (go/go and go/no-go) (Figure 1).

#### The training series

The training series is not graded, it serves as a short training session and gives the experimenter confidence that the child understands what is required of him. Thus, eight circles of different colours (green, red, blue and yellow) were presented on the computer screen at regular intervals one by one. The subject was asked to press a key on the computer when any stimulus appeared. The training series lasted 10 s. Within this series, the experimenter made sure that the child understood the instruction and followed it.

#### Go/go series

Stimuli—circles of different colours—were presented on the screen one after another. The subject’s task was to respond to each stimulus by pressing a key. In other words, a conditioned response to the stimulus was developed in the go/go series. However, since the stimulus flow of the series had a fractal structure. This allows us to exclude the development of a reflex for time. The series lasts 3 min 28 s, includes the presentation of 128 stimuli with different time intervals—circles of different colours (green, red, blue and yellow) and consists of two identical parts. This series evaluates reaction time and the number of missed stimuli in the first part and second part of the series.

#### Go/no-go series

In the go/no-go series the instruction was changed: a response to a certain stimulus (red circles) was prohibited. In other words, the subject needs to inhibit the reaction that he has just developed. The number of erroneous presses on the prohibited stimulus was considered as a value inverse to inhibitory control. Since stimulus flow had a fractal structure again, This allows to exclude the development of a reflex for time, which could change the severity of inhibitory control. The series lasts 3 min 28 s, includes the presentation of 128 stimuli with different time intervals—circles of different colours (green, red, blue and yellow) and consists of two identical parts. Go/no-go series evaluates reaction time, the number of missed stimuli and the number of errors in the first part and second part of the series. In addition, the stability of the inhibition task was assessed by comparing the number of erroneous presses in the second and first parts of the series, which made it possible to identify the influence of fatigue and resource deficiency.

### Visual–spatial working memory

**Figure 2:**
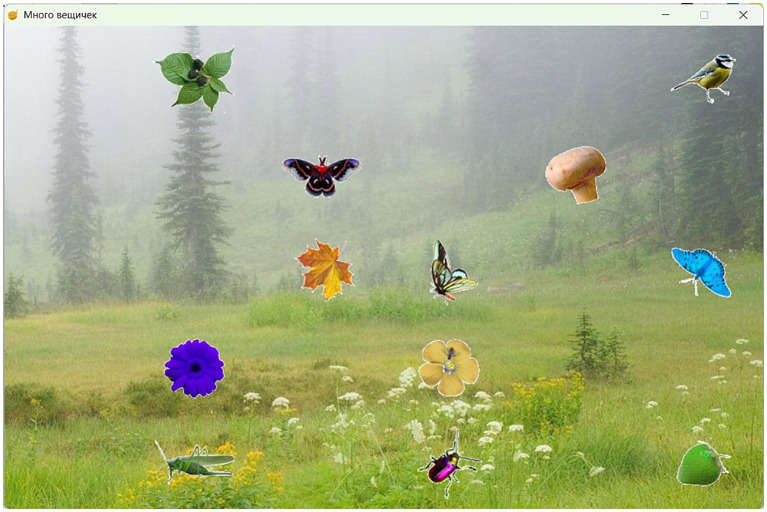
Screen shot of one of the series of the visual–spatial working memory.

Parameters of working memory were assessed using the Visual Working Memory Test (Rasumnikova and Savinich, 2016). The test included three series in which the same set of 30 stimuli was repeated three times in different sequences. The subject’s task was to select a new stimulus each time, i.e., one that had not been selected previously in the current series (but possibly selected in another series). The subject confirmed the choice by pressing the selected stimulus. In other words, the subject had to remember his previous choices in order to select the stimulus that had not yet been selected in the current series from an constantly increasing number of stimuli. If an error was made, i.e., a repeated choice of a stimulus in the current series, the series ended and the next series was offered to begin. The capacity of visual–spatial working memory, equal to the number of remembered stimuli, was assessed in three series (Figure 2).

It is known that information retrieval activates working memory mechanisms, one of which is retrieval-based learning (RBL) (Karpicke, 2017; Conway and Engle, 1994), i.e., improvement of retrieval as the task related to working memory is performed. In this method, retrieval is diagnosed by the number of added stimuli in subsequent series, that is, how much more the subject remembered in the current series compared to the previous one (the best result is used for the assessment).

### Procedure

The study took place in the home of the family where the child lived. This means that data collection took place in a field format with a researcher visiting each family, which has several advantages. Firstly, this format was convenient for the subjects in terms of not having to visit the laboratory, which facilitated the recruitment of the required number of participants. Secondly, this is a step towards resolving the methodological problem of assessing executive functions in a more ecologically relevant environment (Borgnis et al., 2022; Chan et al., 2008). Thirdly, as a consequence, being in a familiar home environment, the children were subject to less stress as a result of participating in unfamiliar procedures, which was reflected in the test results.

Both substitute and biological families were recruited thanks to non-governmental leaders of local parent communities in the city of Nizhny Novgorod, who disseminated information about the research project (mostly in internal online chats or email newsletters). Parents received several benefits from participation that influenced their motivation to open the doors of their home for a visit by the researcher.

On the day of the researcher’s visit to the family, informed consent was obtained from each mother for the child to participate in the study. Each child was told about all the tasks and then asked for verbal consent to participate. The testing procedure was organised in the format of a game quest, where the child had to cope with tasks and move along the route sheet, marking the completed tasks with a selected sticker. This allowed the child to leave the most positive impression of participation in the study. In addition, as a reward, each child was given a small gift, including items for creativity.

### Statistical analysis

All data were assessed by the normality test using the Shapiro–Wilk (W) test and the homogeneity test of variances using the Levene. Depending on the result, parametric or nonparametric tests were used. Since the sample required age control in each analysis due to the wide range, ANCOVA analysis was chosen for unpaired comparisons. In addition, this method allows for the inclusion of additional control variables such as duration of placement or staying with a substitute family. For post-hoc comparisons, the Scheffe test was used for nonparametric data and the Tukey test for parametric data. For correlation analysis, Spearman’s partial correlation was used, controlling for age and duration of institutionalisation history. In analysing the effect of age of placement in an institution, a binary approach was used because the comparison group did not have relevant experience. The analysis of the collected data was performed using the jamovi 2.3.28 software package (Richardson and Machan, 2021).

## Results

### Ever-institutionalised children

The first question addressed was whether children with a history of institutionalisation performed differently on these measures of working memory and inhibitory control than never institutionalised children. Results of the performance measures for the visuospatial working memory and inhibitory control tasks are presented in Table 2. Ever-institutionalised children performed significantly worse on nine of the 11 inhibitory control task measures and on all working memory measures.

**Table 2:** Outcome measures ever-institutionalised group and never-institutionalised group.

Test	Outcome measure	Never-institutionalised mean ± SD (*n* = 66)	Ever-institutionalised mean ± SD (*n* = 64)	Statistics ANCOVA
Inhibition task	Reaction time go/go series the 1st part (mls)	393 ± 69	418 ± 104**	df = 127; *F* = 10.9; *p* = 0.001; *η*^2^ = 0.06
	Reaction time go/go series the 2nd part (mls)	417 ± 82	423 ± 94	df = 127; *F* = 3.34; *p* = 0.07
	Reaction time go/no-go series the 1st part (mls)	500 ± 97	510 ± 125*	df = 127; *F* = 6.15; *p* = 0.014; *η*^2^ = 0.03
	Reaction time go/no-go series the 2nd part (mls)	496 ± 87	521 ± 139**	df = 127; *F* = 9.47; *p* = 0.003; *η*^2^ = 0.05
	Number of missed stimuli go/go series the 1st part	5.32 ± 8.19	7.42 ± 7.15**	df = 127; *F* = 7.56; *p* = 0.007; *η*^2^ = 0.05
	Number of missed stimuli go/go series 2 part	5.62 ± 6.48	9.00 ± 11.4**	df = 127; *F* = 10.4; *p* = 0.002; *η*^2^ = 0.07
	Number of missed stimuli go/no-go series the 1st part	8.02 ± 8.48	11.3 ± 7.81***	df = 127; *F* = 12.6; *p* < 0.001; *η*^2^ = 0.08
	Number of missed stimuli go/no-go series 2 part	8.03 ± 9.84	11.9 ± 13.9**	df = 127; *F* = 9.56; *p* = 0.002; *η*^2^ = 0.06
	Number of errors go/no-go series the 1st part	10.8 ± 3.85	11.4 ± 4.21	df = 127; *F* = 0.08; *p* = 0.8
	Number of errors go/no-go series the 2nd part	10.8 ± 4.03	12.7 ± 4.50*	df = 127; *F* = 6.92; *p* = 0.01; *η*^2^ = 0.05
	Stability of the go/no-go series	0.5 ± 0.5	0.3 ± 0.5**	df = 127; *F* = 7.81; *p* = 0.006; *η*^2^ = 0.06
Visual–spatial working memory	The 1st series	15.1 ± 7.9	12.7 ± 7.1*	df = 127; *F* = 6.45; *p* = 0.01; *η*^2^ = 0.05
	The 2nd series	9.4 ± 5.0	7.9 ± 4.6*	df = 127; *F* = 4.03; *p* = 0.04; *η*^2^ = 0.03
	The 3rd series	10.2 ± 6.2	7.1 ± 4.2***	df = 127; *F* = 12.55; *p* < 0.001; *η*^2^ = 0.09
	Retrieval-based learning	4.1 ± 5.3	2.4 ± 3.8*	df = 127; *F* = 5.38; *p* = 0.022; *η*^2^ = 0.04

The results of multiple linear regression were used to further examine the influence of institutionalisation history on outcomes (number of months spent in an institution). Since the sample has a wide age range, it is expected that the child’s age would be a significant predictor. In this regard, it seemed most valuable to examine the possible exclusive influence of institutionalisation history after controlling for age, as well as the significant interaction between these two markers. After controlling for current age, institutionalisation history remained a significant predictor for the three measures of visuospatial working memory (Table 3). In only one case out of three (WM capacity in the third series) was the length of institutionalisation history the only significant predictor of outcome.

**Table 3:** Regression on memory outcomes in all subjects.

	WM the 1st series			WM the 2nd series			WM the 3rd series			Retrieval-based learning		
	*β*	SE	*p*	*β*	SE	*p*	*β*	SE	*p*	*β*	SE	*p*
History of institutionalisation (months)	−0.123	0.05	0.019	−0.001	0.03	0.97	−0.09	0.03	0.011	−0.007	0.003	0.020
Age of child (years)	0.994	0.34	0.004	0.18	0.22	0.42	0.29	0.25	0.25	0.05	0.02	0.04
*R* ^2^	0.08			0.005			0.05			0.06		
*F*	5.61			0.33			3.43			3.95		

As for the results of the inhibition task, all time characteristics were explained by the age variance of the sample. Both current age and institutionalisation history did not significantly predict the results of the error rate (pressing the forbidden stimulus). However, the stability of the go/no-go series was found to be under the exclusive influence of the duration of institutionalisation experience after controlling for age. Age variance also did not exclude the significance of the contribution of institutional experience to the number of missed stimuli (Table 4).

**Table 4:** Regression on inhibition task in all subjects.

	Number of missed stimuli go/go series 2 part			Number of missed stimuli go/no-go series the 1st part			Number of missed stimuli go/no-go series 2 part			Stability of the go/no-go series		
	*β*	SE	*p*	*β*	SE	*p*	*β*	SE	*p*	*β*	SE	*p*
History of institutionalisation (months)	0.15	0.06	0.01	0.22	0.05	<0.001	0.17	0.08	0.037	−0.01	0.003	0.004
Age of child (years)	−1.76	0.40	<0.001	−1.78	0.34	<0.001	−2.42	0.52	<0.001	0.03	0.022	0.15
*R* ^2^	0.14			0.22			0.15			0.07		
*F*	10.1			17.5			11.0			4.56		

### Substitute care intervention

At the time of the study, all institutionalised children had already been placed in substitute families (mean 48.6 ± 25.1 months). Since the protective factor of substitute care had already been demonstrated in a Russian sample, but these effects mainly concerned the psychological wellbeing of children (McCall et al., 2016), we therefore asked how duration of substitute care contributes to outcomes of working memory and inhibition. The results of multiple linear regression for only the ever-institutionalised group showed that only in one case (the number of missed stimuli in the go/no-go series) did duration of staying in the substitute family remain a significant predictor (Table 5).

**Table 5:** Regression on inhibition task in only ever-institutionalised group.

	Number of missed stimuli go/no-go series the 1st part				
	*β*	SE	*p*	*R* ^2^	*F*
Staying in a substitute family (months)	−0.07	0.03	0.047	0.22	8.40
Age of child (years)	−1.29	0.44	0.004		

After that we returned to the differences between the ever-institutionalised and never institutionalised groups (Table 2) and reran the ANCOVA analysis, but with the additional control of duration of substitute care (number of months of staying in a substitute family). After accounting for the duration of substitute care and the children’s current age, the presence of an institutional history was less significant, but it still influenced on lower measures on visual–spatial working memory in the third series of the test (*F* = 5.3; *p* = 0.02; *η*^2^ = 0.04) and on retrieval-based learning (*F* = 7.7; *p* = 0.006; *η*^2^ = 0.06). For the inhibition task, controlling for the duration of substitute care eliminated any significant differences in the number of errors, and attenuated but saved the significance of differences or reduced the effect sizes for other measures, with the exception of the number of missed stimuli in the first part of the no-go series where the reliability remained unchanged. This may mean that despite the duration of substitute care, the presence of an institutional history still affects some measures of the working memory and inhibition tasks (Table 6).

**Table 6:** Outcomes of measuring of the inhibition task taking into account the duration of substitution care (number of months of staying in a substitute family).

Outcomes measure of the inhibition task	Statistics ANCOVA
Reaction time go series the 1st part (mls)	*F* = 6.7; *p* = 0.01; *η*^2^ = 0.04
Reaction time no-go series the 1st part (mls)	*F* = 6.1; *p* = 0.02; *η*^2^ = 0.03
Reaction time no-go series the 2nd part (mls)	*F* = 7.2; *p* = 0.008; *η*^2^ = 0.04
Number of missed stimuli go series the 1st part	*F* = 5.1; *p* = 0.03; *η*^2^ = 0.03
Number of missed stimuli go series 2 part	*F* = 8.9; *p* = 0.003; *η*^2^ = 0.06
Number of missed stimuli no-go series the 1st part	*F* = 12.9; *p* < 0.001; *η*^2^ = 0.08
Number of missed stimuli no-go series 2 part	*F* = 6.2; *p* = 0.01; *η*^2^ = 0.04
Stability of the go/no-go series	*F* = 7.1; *p* = 0.009; *η*^2^ = 0.05

### Age of placement

The following analyses examined the possible role of age at placement in an institution. Ever-institutionalised children were divided into two groups (early/late) based on age at institutionalisation at 6-month intervals; that is, those placed before 6 months were compared with those placed after 6 months, those placed before or after 12 months, before or after 18 months, and before or after 24 months, that is, within the first 2 years of life. ANCOVAs controlling for current age and length of institutionalisation history were not significant for memory or inhibition outcomes for any of the four (before/after 6, 12, 18, and 24 months) comparisons within the ever-institutionalised group. Spearman partial correlations controlling for age and institutionalisation history were conducted to examine the relationship between age at institutionalisation and memory and inhibition outcomes. Among children with a history of institutional care, no significant correlations were found between any key outcome measures and age at institutionalisation.

We asked whether the outcome measures of the never institutionalised group differed from the early and late institutionalised group. We used ANCOVA, controlling for the child’s current age and length of institutionalisation history, to compare the never institutionalised group with the early and late institutionalised groups, with age at placement at 6, 12, 18, and 24 months as the factor.

**Figure 3:**
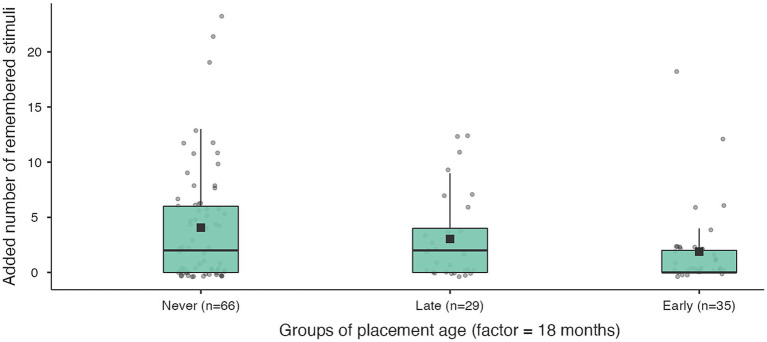
Working memory capacity in the 3rd series for never institutionalised, early institutionalised and late institutionalised groups. Never institutionalised group (mean 10.2 ± 6.2), late institutionalised group (mean 8.0 ± 4.7), early institutionalised group (mean 6.3 ± 3.7). The differences are significant only when comparing never institutionalised and early institutionalised groups (*t* = 2.66; *p* = 0.032).

**Figure 4:**
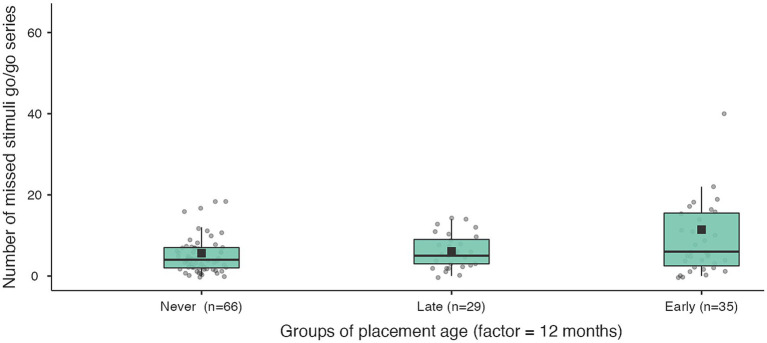
Retrieval-based learning in working memory for never institutionalised, early institutionalised and late institutionalised groups. Never institutionalised group (mean 4.1 ± 5.3), late institutionalised group (mean 3.0 ± 3.9), early institutionalised group (mean 1.9 ± 3.7). The differences are significant only when comparing never institutionalised and early institutionalised groups (*t* = 2.41; *p* = 0.046).

We found that the key working memory outcomes were most significant when comparing the never institutionalised group with those who entered care before 18 months (Table 7; Figures 3, 4). For comparisons with younger placement ages (6 and 12 months) or older placement ages (24 months or younger) the differences in key working memory outcomes were either not significant (for WM in the third series) or the post hoc comparisons (for retrieval-based learning) were significant for both the early and late institutionalised groups.

**Table 7:** Key outcome measures of working memory in never-institutionalised group and late-or early-institutionalised groups (factor = 18 months).

Factor (before and after 18 months)			WM in the 3rd series				RBL			
			Dif	SE	df	Scheffe test	Dif	SE	df	Scheffe test
Never	–	Late	2.11	1.43	125	0.34	1.93	1.23	125	0.07
	–	Early	3.45	1.30	125	0.032	2.70	1.12	125	0.046
Late	–	Early	1.34	1.39	125	0.63	0.77	1.21	125	0.75
			*F* = 3.57; *p* = 0.031; *η*^2^ = 0.05				*F* = 3.00; *p* = 0.041; *η*^2^ = 0.05			

WM, working memory; RBL, retrieval-based learning.

Late (*n* = 29; age of placement 49.3 ± 30.6, months; duration of placement 16.0 ± 19.4 months; staying in substitute care 42.1 ± 25.4 months).

Early (*n* = 35; age of placement 6.9 ± 10.6, months; duration of placement 14.6 ± 10.4 months; staying in substitute care 54.0 ± 23.8 months).

There are no significant differences in the duration of placement in either an institution (*U* = 44; *p* = 0.35) or a substitute family (*t* = −1.94; *p* = 0.058).

**Figure 5:**
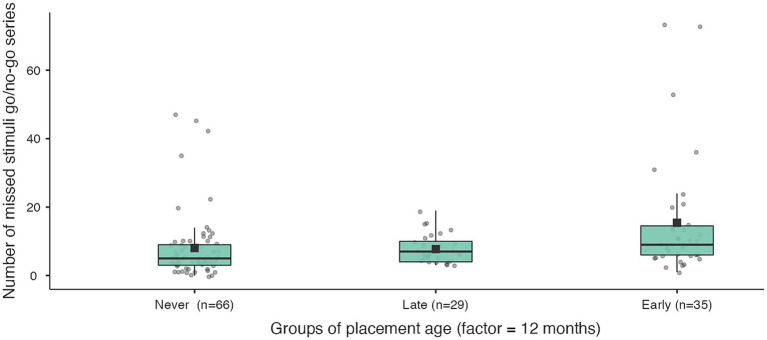
Number of errors in measuring inhibitory control for never institutionalised, early institutionalised and late institutionalised groups. Never institutionalised group (mean 10.8 ± 4.03), late institutionalised group (mean 12.0 ± 4.4), early institutionalised group (mean 13.4 ± 4.6). The differences are significant only when comparing never institutionalised and early institutionalised groups (*t* = −2.49; *p* = 0.037).

**Figure 6:**
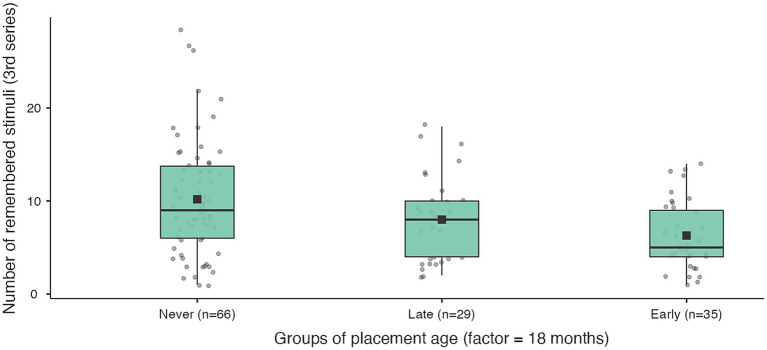
Number of missed stimuli go/go series in the 2nd part for never institutionalised, early institutionalised and late institutionalised groups. Never institutionalised group (mean 5.6 ± 6.5), late institutionalised group (mean 6.1 ± 4.2), early institutionalised group (mean 11.4 ± 14.6). The differences are significant only when comparing never institutionalised and early institutionalised groups (*t* = −2.52; *p* = 0.045).

**Figure 7:**
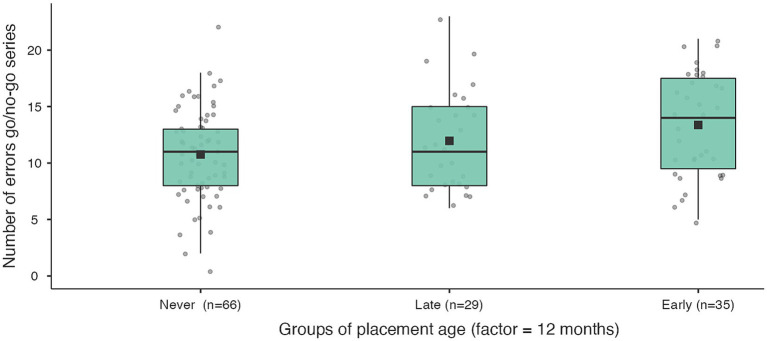
Number of missed stimuli go/no-go series in the 2nd part for never institutionalised, early institutionalised and late institutionalised groups. Never institutionalised group (mean 8.0 ± 9.8), late institutionalised group (mean 7.7 ± 4.2), early institutionalised group (mean 15.4 ± 17.8). The differences are significant only when comparing never institutionalised and early institutionalised groups (*t* = −2.65; *p* = 0.033).

The key outcome of the inhibition task (number of errors), after controlling for the child’s age and the duration of institutional experience, is most significant in the only comparison of the never institutionalised group with the institutionalised group before 12 months. In addition, never institutionalised children omit significantly fewer stimuli than early institutionalised children (before 12 months) in the second part of both experimental series (Table 8; Figures 5–7). When compared with groups whose age of placement is even younger (before 6 months), the significance of the difference in the number of omissions increases, and decreases when compared with later ages of placement (before 18 and 24 months).

**Table 8:** Outcome measures of inhibition task in never-institutionalised group and late-or early-institutionalised groups.

Factor (before and after 12 months)			Number of errors go/no-go series the 2nd part				Number of missed stimuli go/go series the 2nd part				Number of missed stimuli go/no-go series the 2nd part			
			Dif	SE	df	Tukey test	Dif	SE	df	Scheffe test	Dif	SE	df	Scheffe test
Never	–	Late	−1.15	1.16	125	0.58	−2.53	2.32	125	0.55	−3.10	2.99	125	0.59
	–	Early	−2.59	1.04	125	0.037	−5.24	2.08	125	0.045	−7.08	2.68	125	0.033
Late	–	Early	−1.43	1.13	125	0.415	−2.71	2.27	125	0.46	−3.98	2.92	125	0.40
			*F* = 3.13; *p* = 0.047; *η*^2^ = 0.05				*F* = 3.18; *p* = 0.045; *η*^2^ = 0.04				*F* = 3.53; *p* = 0.032; *η*^2^ = 0.05			

Late (*n* = 29; age of placement 30.7 ± 4.49, months; duration of placement 16.3 ± 19.2 months; staying in substitute care 39.6 ± 25.2 months).

Early (*n* = 35; age of placement 4.49 ± 4.65, months; duration of placement 14.3 ± 10.7 months; staying in substitute care 56.1 ± 22.7 months).

The difference is not significant for the duration of placement in the institution (*U* = 46; *p* = 0.54), but the early institutionalised group stayed in the substitute family significantly longer (*t* = −2.8; *p* = 0.008).

## Discussion

The aim of the research reported here was to explore the impact of early institutionalisation history on the formation of inhibitory control and working memory in children, and to test the speculative hypothesis if there is a critical period in the development of these functions which is limited to the first 18 months of the child’s life. This hypothesis was based on the dependence of various components of the formation of executive functions on the development of neural networks generated by the prefrontal cortex (Hanson et al., 2013; Cheng et al., 2021; Teicher et al., 2016). Interest in executive functions is related to their resource component. It is understood that executive functions are responsible for activity associated with behavioural change, i.e., numerous types of activity that allow a child traumatised in early childhood to restore their potential in the cognitive sphere. We hypothesise that a decrease in the specialisation of neural networks during stress in early ontogenesis (Malave et al., 2022; Hambrick et al., 2019; Hostinar et al., 2018) reduces the potential for recovery of executive functions at a later age.

The development of executive functions is usually associated with the so-called “expectable environment” (Nelson and Gabard-Durnam, 2020; Fox et al., 2010), which is rich in sensory and cognitive stimulation. It has been established that executive functions are formed in preschool childhood (Best et al., 2009; Rueda et al., 2016), with working memory developing significantly already in the first 12 months of life (Buss et al., 2018) and by the age of six, reached a basic modular structure (Gathercole et al., 2004), while inhibitory control much more slowly (Nikolaeva et al., 2020), nevertheless is observed by age of seven the relative level of productivity of this component (Best et al., 2009; Rueda et al., 2016). However, when the environment does not match the expected experience, the development trajectories of the EF may vary (Sonuga-Barke et al., 2017; Vasilyeva et al., 2017).

We first compared outcomes of measures inhibition and working memory tasks between the never institutionalised group and the ever-institutionalised group living in substitute care at the time of the study. The ever-institutionalised group had the lowest scores on most outcomes, including number of errors and number of stimuli remembered. These findings are consistent with previous studies of working memory (Bos et al., 2009) and inhibitory control (McDermott et al., 2013; Loman et al., 2013) in children with institutional history. Analysing the role of substitute care, we found that the protective effect of family was significant for the working memory task, eliminating any differences between the ever and never institutionalised groups. However, the significant differences in performance on the inhibition task remained. A previous experiment compared the outcomes of never institutionalised children with two groups of children who entered early institutional care (in the first 2–3 months of life), with the only difference being that some remained in institutional care and the others were immediately placed in foster care (McDermott et al., 2013). It was shown that after 8 years, children from both foster care and institutional care were equally worse on the inhibition task compared with a control group that had never been institutionalised. In other words, it seems that the factor of early placement in an institution, even for a short time, was more influential than the early intervention of foster families. It appears the rule “the earlier the better” (Zeanah et al., 2011) (implying fostering) helps, but does not solve, all the developmental challenges of children placed in institutions.

Then, in accordance with the objectives of this study, the ever-institutionalised children were divided into two groups: early and late institutionalised. The empirical basis for the age cutoff was previous research showing that children institutionalised after 18 months have better cognitive development outcomes after institutional intervention (Hawk et al., 2018), in another study this cutoff was lowered to 9 months (Julian, 2013), and in the case of severe deprivation observed in Romanian institutionalised children—to 6 months (Rutter, 2006). Since this may mean the most sensitive age cutoff for working memory and inhibitory control varies somewhere within the first 2 years of life, we tested different cutoffs in 6-month increments within 24 months. Thus, we compared groups of those institutionalised before 6 months with those after this age, then those institutionalised before 12 months with those after, and so on.

Although the analysis of the results depending on the age of placement only within the ever-institutionalised group did not show significant differences, they were found when comparing the early institutionalised groups with the never institutionalised group. For the performance of working memory tasks, the most sensitive age cutoff was 18 months, while for inhibition tasks it was 12 months. This means that the never institutionalised group had significantly higher scores on the working memory key test tasks compared only with those children who were institutionalised before 18 months, whereas there was little difference in scores with the group institutionalised after this age.

For the inhibition scores, the age cutoff dropped to 12 months, moreover differences appear in the second part of the go/no-go series. This can indicate greater vulnerability and instability of inhibitory control due to the lack of resources to support more complex cognitive activity that is consistent with the literature on the disruption of neural networks in children exposed to ACE’s in the early period of development (Nelson et al., 2023). This is particularly curious given that the early and late institutionalised groups did not differ in gender, caregiver characteristics, or length of institutionalisation, but the early institutionalised group received more substitute care, and in the comparison group at the 12-month cutoff, this difference in duration of substitute care was statistically significant.

One of the results of the study was the discovery of vulnerability to stress in the early ontogenesis of the retrieval-based learning mechanism in working memory, which is responsible for resisting the mechanism of proactive interference (Anderson et al., 2000). This mechanism allows the retention of information, despite the emergence of new information (Karpicke, 2012). In the current study, working memory is assessed in three attempts, each of which presents the same set of stimuli, but in a different sequence. The child is required to select a stimulus that was not selected in the current attempt, but could have been selected in the previous attempt. When the child makes the first attempt—the first reproduction, they often remember many stimuli. Therefore, when they reproduce them for the second time, they encounter proactive interference, when the previous information interferes with the reproduction of the next one (Anderson et al., 2000). Most normally developing children after 5 years of age try to find a method to resist such a mixture of stimuli and strive to develop a strategy for independently memorising information in the third reproduction (Forest and Amso, 2023). But institutionalised children, probably, are not ready to resist this mechanism of projective interference, and therefore the last reproduction has a minimum number of elements.

The suppression of irrelevant information is a function of the prefrontal cortex (PFC) (Anderson and Hulbert, 2021). The specificity of its formation is that throughout a child’s development it is involved in the process of developmental management (Andersen, 2003) beginning in the early postnatal period. Myelination in the PFC differs from that occurring in other brain regions in the postnatal period due to the longer presence of premyelinating oligodendrocytes (remaining from the prenatal period). They are more vulnerable to perinatal damage than mature oligodendrocytes and predominate in the frontal lobe areas at birth (Back et al., 2001), which clearly makes the early white matter of the frontal lobe susceptible to injury. It is worth emphasising the important role of early migration of prefrontal neurons to the cortex: this allows them to participate in the organisation of early brain activity (Collin and van den Heuvel, 2013), but at the same time leads to a unique vulnerability of the frontal white matter to early injury (Hodel, 2018). Modern non-invasive methods of studying infants convincingly demonstrate that it is in the first years of life that extensive structural development of the prefrontal cortex occurs, and it plays a unique role in connecting other brain structures to the general network (Buss et al., 2018; Massera et al., 2023; Wade et al., 2022; Kolb et al., 2012).

The results of this study show that the outcomes of working memory and inhibitory control of children who were placed in an orphanage after 18 months, but later found themselves in a substitute family, are comparable to the outcomes of children without the experience of institutionalisation. However, such a correspondence is not observed in children who were placed in an orphanage before 18 months. In addition, early institutionalised children ended up spending more time in the substitute family than late institutionalised children. However, the beneficial effect of family placement on cognitive functions was more successful in late institutionalised children. The “earlier the better” rule probably does not apply to the sample of this study as well, since some of the early institutionalised children were placed to substitute families 2–3 months after entering an institution, but this did not affect their results. Could these data serve as a prerequisite for the presence of a critical period in the formation of working memory and inhibitory control?

The term “critical period” comes from animal models [e.g., (Hubel and Wiesel, 1970)], is commonly used to describe sensory systems [e.g., (Kral et al., 2019; Maurer et al., 2007)], and explains how experience affects the brain in ways that permanently alter performance. In humans, the term “sensitive periods” is more commonly used to reflect the brain’s characteristic neuroplasticity and high sensitivity to the environment throughout life (Zeanah et al., 2011; Stamps and Luttbeg, 2022; Frankenhuis and Walasek, 2020). We propose to consider the concept of “critical” period in relation to the development of working memory and inhibitory control in the first 18 months of life, underlining the neurobiological nature of the phenomenon (Larsen and Luna, 2018; Uylings, 2006). It has been suggested the plastic capabilities of the brain are provided by the absence of myelination. Gradual myelination leads to a narrowing of these capabilities (Uylings, 2006). A recent intriguing study showed that in 29 institutionalised infants, some methylated genes were correlated with the length of stay in an orphanage, including a gene required for the organisation of myelinated axons during brain development (Naumova et al., 2019). In other words, by using the term “critical” period, we want to emphasise that after being institutionalised, some children adjust worse than others to the new conditions (Ellis et al., 2009), and, different brain systems within a person may adjust to new conditions at different rates (Zeanah et al., 2011).

We would like to acknowledge several important limitations of this study that should be taken into account in the interpretation. The first and most important limitation is related to the wide age range of the study sample. Despite constant age control in statistical analyses, the results remain vulnerable to proof. In addition, the lack of information on the prenatal period of children’s development and the quality of parent–child relationships with their biological parents in the first 18 months of life also limits the possibilities of interpretation. Finally, the present study is cross-sectional, which precludes claims about causal mechanisms for the observed results. Further development of the concept of a critical period for the development of executive functioning components requires further longitudinal studies with strict controls for the life trajectory of the child exposed to adversity.

In conclusion, when looking for a common denominator that may help explain the impact of family deprivation during early life critical periods in brain development on infants placed in institutions the lack of, primarily maternal, nurturing care, is posited here as a major candidate for the source of such deprivation. Maternal tactile affection, as mediated by gentle caressing touch, provides an optimal stimulus for a class of thermo-mechanosensitive unmyelinated afferents innervating the skin of the body, called c-tactile afferents (CT) (McGlone et al., 2014). Animal models of juvenile social isolation result in myelination changes that mimic conditions related to neurodevelopmental disorders, with prolonged social isolation, and therefore tactile deprivation, inducing hypomyelination in the PFC, partially explaining the long-term consequences of early childhood experiences on the development of working memory and inhibitory control reported in this paper (Liu et al., 2012).

## Data Availability

The raw data supporting the conclusions of this article will be made available by the authors without undue reservation.
